# Long-Term Follow-Up of Mechanical Circulatory Support in Peripartum Cardiomyopathy (PPCM) Refractory to Medical Management: A Multicenter Study

**DOI:** 10.3390/life12010087

**Published:** 2022-01-07

**Authors:** Khalil Jawad, Alex Koziarz, Maja-Theresa Dieterlen, Jens Garbade, Christian D. Etz, Diyar Saeed, Elena Langer, Holger Stepan, Ute Scholz, Michael Krause, Paolo Brenner, Uwe Schulz, Michael A. Borger, Sandra Eifert

**Affiliations:** 1 Leipzig Heart Center Leipzig, University Clinic of Cardiac Surgery, 04289 Leipzig, Germany; MDieterlen@web.de (M.-T.D.); jens.garbade@gesundheitnord.de (J.G.); christian.etz@helios-gesundheit.de (C.D.E.); Diyar.Saeed@helios-gesundheit.de (D.S.); uwe.schulz@helios-gesundheit.de (U.S.); Michael.Borger@helios-gesundheit.de (M.A.B.); Sandra.Eifert@helios-gesundheit.de (S.E.); 2 Peter Munk Cardiac Center, University of Toronto, Cardiac Surgery, Toronto, ON M5S 1A1, Canada; alexkoziarz@gmail.com; 3 Department of Obstetrics, University of Leipzig, 04109 Leipzig, Germany; Elena.Langer@medizin.uni-leipzig.de (E.L.); Holger.Stepan@medizin.uni-leipzig.de (H.S.); 4 Centre of Coagulation Disorders, 04109 Leipzig, Germany; u.scholz@labor-leipzig.de (U.S.); m.krause@labor-leipzig.de (M.K.); 5 Department of Cardiac Surgery, University of Munich, 80539 Munich, Germany; paolo.brenner@med.uni-muenchen.de

**Keywords:** peripartum cardiomyopathy, heart failure, cardiac surgery, left ventricular assist device, heart transplant

## Abstract

Background: Peripartum cardiomyopathy (PPCM) is a rare, life-threatening form of heart disease, frequently associated with gene alterations and, in some cases, presenting with advanced heart failure. Little is known about ventricular assist device (VAD) implantation in severe PPCM cases. We describe long-term follow-up of PPCM patients who were resistant to medical therapy and received mechanical circulatory support or heart transplant. Methods and results: A total of 13 patients were included with mean follow-up of eight years. Mean age of PPCM onset was 33.7 ± 7.7 years. All patients were initially treated with angiotensin-converting enzyme inhibitors and beta-blockers, and four received bromocriptine. Overall, five patients received VADs (three biventricular, two isolated left ventricular) at median 27 days (range: 3 to 150) following childbirth. Two patients developed drive line infection. Due to the short support time, none of those patients had a stroke or VAD thrombosis. In total, five patients underwent heart transplantation, of which four previously had implanted VADs. Median time to transplantation from PPCM onset was 140 days (range: 43 to 776), and time to transplantation from VAD implantation were 7, 40, 132, and 735 days, respectively. All patients survived until most recent follow up, with the exception of one patient who died following unrelated abdominal surgery two years after PPCM recovery. Conclusions: In patients with severe, life-threatening PPCM refractory to medical management, mechanical circulatory support with or without heart transplantation is a safe therapeutic option.

## 1. Introduction

Pregnancy is a multidimensional condition; previously healthy women may develop diseases during pregnancy, and women with pre-existing disease fall pregnant. These conditions require specialized treatments and should be managed in a multidisciplinary approach [1,2]. Disease management must be optimized, since optimal treatment for the mother may harm the fetus and treatment of the fetus may harm the mother.

During pregnancy, several mechanisms are thought to be responsible for why the mother does not reject the fetus. These are categorized as fetal factors, such as trophoblast cell properties and altered MHC Class I expression, as well as local maternal factors such as uterine natural killer cells and shifted T-helper cell cytokines type II. Persisting fetal cells in the maternal circulation may have implications on the development of autoimmune diseases.

Peri- and post-partum cardiomyopathy (PPCM) is a rare but life-threatening condition of an autoimmune etiology [3]. The main pathomechanism was originally discovered by Hilfiker-Kleiner, who reported the existence of an oxidative stress-mediated 16-kDa fragment known as cathepsin D [4]. Cleavage of cathepsin D from the nursing hormone prolactin expresses a highly cardiotoxic effect. Accordingly, this may lead to microcirculatory and metabolic changes in cardiomyocytes, which have been shown to be reversible [5]. Further discovery by Hilfiker-Kleiner demonstrated that female mice with cardiomyocyte-specific deficiency of the pro-apoptotic and anti-angiogenic transcription factor STAT3 developed PPCM [4].

PPCM presents with acute onset heart failure during the third trimester of pregnancy and up to six months postpartum. The estimated incidence is 1 in 1000 births worldwide [6]. Inhibition of prolactin secretion by bromocriptine, a dopamine D2 receptor agonist, is part of a common treatment regimen known as BOARD (Bromocriptine, Oral heart failure therapies, anticoagulants, vaso-relaxing agents, and diuretics, as proposed by the German Network for PPCM) [7]. This therapy has been supported as an addition to the optimized standard heart failure therapy offered [7]. In severe cases, refractory to medical management and surgical options such as mechanical circulatory support or heart transplantation may be offered. However, evidence is limited to case reports with no long-term term follow-up studies published [8]. Therefore, we report on a cohort of women who presented with severe PPCM and evaluate the long-term safety of mechanical circulatory support and heart transplantation. 

## 2. Methods

We performed a multicenter retrospective cohort study of women presenting with PPCM requiring mechanical circulatory support or heart transplantation. All women were managed at two high-volume cardiac surgery centres (Leipzig Heart Center and University of Munich, Munich, Germany). Careful medical history was taken from each patient. Medical and surgical therapies were evaluated in this entity of young females who were otherwise healthy patients. Follow-up data were acquired by reviewing the prospective hospital database results obtained by annual written or telephone interviews. Continuous variables were presented as mean with standard deviation. Categorical variables were presented as counts with percentage. Statistical analyses were performed using IBM SPSS Statistics, version 26 (SPPS Inc., Chicago, IL, USA).

## 3. Results

### 3.1. Demographic Data

Overall, 13 patients with severe PPCM were included. Demographic data is reported in Table 1 for each patient. Mean follow-up was eight (range: 2 to 10) years. All times of PPCM onset were post-partum with a median time of PPCM onset of 16 (range: 1 to 138) days following childbirth. Median age at delivery was 33.7 (±7.7) years. Median BMI was 22 (range: 16.5 to 33.2) kg/m^2^. In total, five of the 13 patients had positive family history of cardiomyopathy. PPCM occurred after second pregnancy in six patients, third pregnancy in four patients, first pregnancy in two patients, and fifth pregnancy in one patient. Anticoagulation was observed tightly; however, two of the five patients with major bleeding required transfusion due to peripartum status. All patients were initially treated with optimal heart failure medication therapy in specialized centers before referral to our surgical center, of which four received bromocriptine after the Protocol of German PPCM Network for 60 days. All patients were initially referred to both centers for advanced heart failure management, including surgical options such as VAD implantation or heart transplantation.

### 3.2. Genetic Investigation of STAT3

Many studies have shown that a mutation in the achromatic gene titin (TTN) plays an important role in the development of PPCM [9]. A genetic cause of the disease has been identified in up to 20% of the patients who developed PPCM [10]. Genetic tests are not routinely performed; however, given the potential complications of PPCM, it is recommended to perform such tests on patients with PPCM, as well as family members who are at risk [11].

In our cohort, genetic investigation revealed a novel mutation in bp 255 (T insertion), exon 17-1 of STAT3 in one patient who was transplanted. All other patients demonstrated known mutations.

### 3.3. Optimal Medical Therapy 

The severity of the disease varies enormously. Patients with mild heart failure symptoms can be treated with oral medications on an outpatient basis. The use of bromocriptine with the standard heart failure medical therapy has demonstrated an improvement in myocardial recovery in both mild and severe cases of PPCM, which may reduce future requirement for MCS or heart transplantation. Nevertheless, some patients were unfortunately unable to recover despite optimal medical therapy [12], necessitating invasive therapy with MCS or heart transplantation. In our cohort, all patients were transferred to Leipzig Heart Center or University of Munich Cardiac Surgery Center in cardiogenic shock (INTERMACS Level 1). In total, eight patients received an aggressive therapy with diuretics and inotropes, and three cases received vasopressors in addition to their optimal medical therapy. These eight patients were able to be discharged after weaning from the intravenous medication.

### 3.4. Electrocardiogram (ECG) and Implantable Cardioverter Defibrillator (ICD)

Studies have not yet shown ECG abnormalities specific to PPCM [13]. ECG findings in patients presenting with PPCM most commonly demonstrate T-wave abnormalities and atrial abnormalities [13]. Compared to age-matched healthy controls, PPCM patients have a higher risk for sudden cardiac death, particularly those with an ejection fraction less than 35%. Therefore, an ICD implantation should be considered. In our cohort, two patients received an ICD due to ventricular tachyarrhythmia directly after PPCM diagnosis to prevent sudden cardiac death. One of the implanted ICDs was explanted during heart transplantation.

### 3.5. Mechanical Circulatory Support

Based on the fact, that the majority of PPCM patients recover after treatment with short-term MCS, long-term MCS is not recommended routinely unless a patient initially presents with life-threatening PPCM. In our cohort, five patients underwent mechanical circulatory support: five patients received ventricular assist devices (three BiVAD, two LVAD—of them, one following VA-ECMO, ECLS) despite aggressive therapy with inotropes and vasopressors at median 27 (range: 3 to 150) days following child birth (Table 2). In our cohort, one patient received an urgent VA-ECMO as bridge to recovery for five days. In this case, we were able to stabilize this patient to INTERMACS level 3, followed by an elective LVAD implantation. After an uneventfully clinical course, we discharged the patient in a stable condition.

### 3.6. Heart Transplantation

In the worst-case scenario, once all possible conservative therapy options do not help, heart transplant is the last treatment choice for these seriously ill patients. Up to 10% of PPCM patients require a heart transplantation [14].

In total, five patients received an orthotopic heart transplantation, of which four previously had implanted ventricular assist device (three BiVAD, one LVAD). Median time to transplantation from PPCM onset was 140 (range: 43 to 776) days, and times to transplantation from ventricular assist device implant were 7, 40, 132, and 735 days.

The immunosuppressive regimens were aligned to the international guidelines. As expected, all transplanted women did not show any transplant rejection in their biopsies.

### 3.7. Patient’s Follow Up for 8 Years

In total, 12 of the 13 patients are alive and in NYHA class I-II and without lack of exercise capacity. All children born under these circumstances are alive and well.

Overall, two patients developed a driveline infection with prove of staphylococcus aureus in microbiological investigation, and one of these patients underwent a heart transplantation two years later. This patient died in November 2019 due to unrelated abdominal plastic surgery two years after heart transplantation after myocardial infarction. The second patient does not meet the transplantation criteria, since the driveline infection was treated with antibiotics.

## 4. Discussion

In this retrospective cohort we analysed the data of 13 women with PPCM who presented to Leipzig Heart Center and Munich University Hospital with cardiogenic shock.

PPCM is a rare but serious and life-threatening condition [15]. PPCM may occur in the last month of pregnancy, during delivery, or in the first six postpartum months, and may have an acute or insidious onset [16].

Multiparity, pre-eclampsia, advanced or early maternal age, prolonged use of beta-agonists, family history, ethnicity, smoking, diabetes, hypertension, and previous incidence of PPCM are risk factors for PPCM. Sliwa and colleagues have shown that genetics play a role in up to 10–15% of women who experience PPCM; however, the penetrance is not 100% among women with PPCM who were genetically and clinically evaluated [17].

The physiology of increased oxidative stress during pregnancy leads to increased formation of reactive oxygen species (ROS) [18]. An increase of total antioxidant capacity imparted by an up-regulated expression of mitochondrial superoxide dismutase 2 (SOD2) usually compensates the increased formation of ROS [18].

STAT3 have an important function in the postpartum maternal heart. STAT3 and other proteins regulate SOD2 after delivering [4].

Female mice with a cardiomyocyte-specific deficiency of STAT3 could develop PPCM phenotypes [4].

In the peripartum phase, is it known that extensive bleeding or a hyperosmolar stress may lead to reduction in cardiac STAT3; nevertheless, there is no strong evidence for genetic factors leading to reduction in cardiac STAT3 expression in PPCM patients [19]. In our cohort, one patient revealed an unknown mutation in bp 255 (T insertion), exon 17-1 of STAT3 who was successfully transplanted.

Early diagnosis and optimization of medical therapy could improve the prognosis in patients suffering from PPCM up to full recovery. 

In our cohort, the rate of patients received bromocriptine was low. Bromocriptine was not necessarily the treatment of choice at this time, although recommended. A multicenter randomized trial evaluating the dopamine D2 agonist bromocriptine for eight weeks in addition to standard heart failure medication reported superior outcomes for acute PPCM patients randomized to single use of bromocriptine [20]. 

Nevertheless, mechanical circulatory support and heart transplant are required for and indicated in selected patients, in whom optimal medical management (OMM) failed. Haghiki and colleagues describe in their study that 50% of patients fully recover after OMM, 35–40% partially recover, and a small number of women persist in severe reduced LV ejection fraction with the need of mechanical circulatory support or heart transplantation [21]. Accordingly, in patients with severe PPCM refractory to medical management mechanical circulatory support with short and long(er) term MCS including ECLS and ventricular assist device, respectively, is a safe and effective management strategy. 

Early diagnosis and early initiation of medical therapy may be associated with good outcome, which has been supported by other studies [22]. However, in cases of right ventricular dysfunction, patient’s recovery is low [23]. The fact, that patients in our cohort were referred to our centers with end-stage heart failure (INTERMACS 1) for possible mechanical circulatory support or heart transplantation is the main reason for our low initial recovery rate.

At the time of admission, all our patients required inotropic medical support. Our survival rate at five years was 100%, and was 92.3% after eight years, with one patient experiencing non-cardiac death following a complication after abdominal surgery. As above mentioned, five patients in our cohort received mechanical circulatory support (three BiVAD, two LVAD), and five patients underwent heart transplantation. The rejection rate or the transplant-related complications in patients who underwent heart transplant due to PPCM showed same results as other populations [24]. Only one patient is still supported by a LVAD up to this date. We were not able to explant it as all follow-up echocardiography did not demonstrate sufficient left ventricular recovery. Boehmer and colleagues reported in his cohort a 50% rate of patients who recovered after mechanical circulatory support implantation and were explanted [25]. The initial medical treatment at the referral hospital failed. The early LVAD implantation in our cohort is based on the fact that these patients were in a very poor cardiac condition at the time of admission.

The survival rate in patients who underwent long-term mechanical circulatory support due to PPCM is higher than those with non-PPCM. This is likely due to their younger age and fewer number of comorbidities [12].

According to the ESC guidelines, ECLS could be initiated (for 14–28 days). In case of no improvement, midterm MCS should be considered earliest this time. LVAD implantation as bridge to recovery or transplantation can be necessary in 2–7% of PPCM patients [1].

The first studies on this topic were case reports that were unable to comment on the pathogenesis and management of PPCM. Since then, remarkable advances have been made in understanding the pathogenesis and identifying beneficial medications. Additionally, drugs such as bromocriptine have been introduced and lead to a significant improvement the long-term outcome and survival rate.

In this retrospective analysis, we demonstrated with long-term follow-up that MCS and heart transplantation are acceptable therapeutic options for patients with cardiogenic shock resistant to medical therapy.

This study had several strengths. Firstly, to our knowledge, it is the largest cohort of patients with PPCM presenting with cardiogenic shock requiring MCS or heart transplantation. Second, genetic analysis was performed in all patients. Third, patients in our cohort were managed surgically in a high-volume cardiac center, which increases the internal validity of our evaluation of the safety and efficacy of MCS and heart transplantation [26]. Notably, in our cohort, the percentage of patients who received long-term MCS or heart transplantation is higher than other studies, as these were outside referrals whereby conservative medical therapy was insufficient. The generalizability of these results is therefore limited to other high volume cardiac surgery centers. Other limitations of this study include the fact that patients were identified retrospectively, so selection bias favoring patients who had an initially better prognosis cannot be ruled out. In addition, our sample size was too small to make conclusions regarding which patient characteristics best predict survival, improved heart function, and quality of life. Furthermore, in our cohort, we did not perform an analysis related to the percentage rate of PPCM patients at our centre or a scoring about quality of life.

## 5. Conclusions

In patients with severe, life-threatening PPCM refractory to medical management, mechanical circulatory support is a safe therapeutic option as well as a highly effective bridge with or without heart transplantation.

## Figures and Tables

**Table 1:** Demographics of patients presenting with severe peripartum cardiomyopathy.

Patient Number	Age at PPCM (Years)	Number Days PPCM Development Following Delivery	BMI (kg/m^2^)	Positive Family History	Parity of Woman at PPCM Development	Follow-Up (Years)
1	25	6	19.7	yes	1	10
2	49	5	27.7	no	5	6
3	28	1	26	yes	3	8
4	24	31	21.2	yes	2	7
5	34	85	16.5	yes	3	10
6	43	18	22	no	2	9
7	34	12	18.4	yes	3	8
8	31	138	23.3	no	2	11
9	31	21	32.5	no	2	6
10	40	7	20.1	no	3	10
11	25	137	33.2	no	1	2
12	33	14	NA	no	2	NA
13	41	NA	NA	no	2	NA

PPCM: peripartum cardiomyopathy; BMI: body mass index.

**Table 2:** Management of patients who underwent mechanical circulatory support or heart transplant.

Patient Number	Administered Bromocriptine	VAD Implant Following PPCM (Days)	VAD Type	Heart Transplant Following PPCM (Days)	Survival at Last Follow-Up	Complications
1	No	3	BiVAD	43	Yes	None
4	No	41	LVAD (HW)	776	Death at 33 years old.	Drive line infection Death following unrelated abdominal surgery.
5	No	8	BiVAD	140	Yes	None
8	Yes		None	130	Yes	None
10	No	150	BiVAD	157	Yes	None
11	Yes	27	LVAD (HW)	None	Yes	Drive line infection

VAD: ventricular assist device; PPCM: peripartum cardiomyopathy; HW: heart ware.

## Data Availability

The data presented in this study are available on request from the corresponding author. The data are not publicly available due to privacy and ethical restrictions.

## Abbreviations

Peripartum cardiomyopathy (PPCM); ventricular assist device (VAD); Interagency Registry for Mechanically Assisted Circulatory Support (INTERMACS).
